# Consequences of early extraction of compromised first permanent molar: a systematic review

**DOI:** 10.1186/s12903-018-0516-4

**Published:** 2018-04-05

**Authors:** Afnan M. Saber, Doua H. Altoukhi, Mariam F. Horaib, Azza A. El-Housseiny, Najlaa M. Alamoudi, Heba J. Sabbagh

**Affiliations:** 1 Pediatric Dentistry Department, Faculty of Dentistry, King Abdulaziz University, P.O. Box 80209, Jeddah, 21589 Saudi Arabia; 2 Pediatric Dentistry Department, Faculty of Dentistry, Alexandria University, Alexandria, Egypt

**Keywords:** First permanent molar, Early extraction, Early loss, Result or effect of extraction, Children

## Abstract

**Background:**

The aim of this study was to systematically review the literature to determine the sequelae of early extraction of compromised first permanent molars (FPMs) with regard to the skeletal and dental development of 5- to 15-year-old children. Meta-analysis was conducted when applicable.

**Methods:**

Our research protocol included a search strategy, inclusion/exclusion criteria, and a data extraction plan. The search engines used were PubMed, Scopus, and Science Direct. Study selection was performed independently by three reviewers. Articles published from 1960 to 2017 were reviewed based on inclusion and exclusion criteria. Meta-analysis was performed to compare space closure between upper and lower arches.

**Results:**

Eleven studies fulfilled the inclusion criteria. The consequences were decrease in post extraction space, accelerated development and eruption of second permanents molars (SPMs) and third molars, a decrease in caries and/or fillings on the proximal surfaces of adjacent teeth, lingual tipping and retrusion of incisors, and counter clockwise rotation of the occlusal plane.

**Conclusion:**

There were several consequences of early extraction of FPMs, which were related to skeletal and dental development. Our systematic review suggests that comprehensive evaluation of the compromised FPMs should be performed before planning an extraction. The ideal time for FPM extraction is when the SPM is at the early bifurcation stage in order to achieve complete closure of the extraction space by the SPM. Benefits should be weighed over the risks to decrease the risk of unfavorable outcomes as much as possible. However, due to the limited evidence on the outcomes and variables that influence them, high-quality prospective studies are needed.

**Electronic supplementary material:**

The online version of this article (10.1186/s12903-018-0516-4) contains supplementary material, which is available to authorized users.

## Background

Dental caries is the most common infectious disease worldwide [1]. Globally, 60%–90% of school children have dental caries [2]. The first permanent molar (FPM) emerges early, so is more prone to dental caries and possible premature extraction before 15 years of age. The importance of this tooth lies in its major role in maintaining normal masticatory function and dentofacial harmony [3].

Many factors should be considered before determining the appropriate treatment method for a badly decayed FPM, such as the level of crown destruction, the degree of pulp maturation, the status of the developing dentition, the severity of dental pain, the attitude of the child’s parent(s), and the patient’s ability to withstand long treatment under local anesthesia. Accordingly, some clinicians favor early extraction of these teeth because they are more likely to have a poor prognosis and require extraction later on [4]. On the other hand, others prefer to restore an extensively decayed FPM. Extraction of FPMs may be highly considered or not depending on treatment consequences and outcomes [5].

In view of the controversy regarding the consequences of early extraction of compromised FPMs, this systematic review of the literature was undertaken to determine the effects and sequelae of early extraction of these teeth with regard to the skeletal and dental development of children aged 5–15 years. Meta-analysis was carried out when applicable.

## Methods

### Literature search

We registered our research topic at the Center for Reviews and Dissemination (registration number CRD42015020275). We developed a research protocol that included a search strategy, inclusion/exclusion criteria, and a data extraction plan. The search started in May 2015 and ended in 2017. The search strategy comprised key words used separately or in various combinations (“first permanent molar tooth”, “early extraction”, “early loss”, “results” and “effects”), and was performed in three search engines (PubMed, Scopus, and Science Direct) from 1960 to 2017. No language restriction was imposed.

### Study selection

Article titles were assessed for eligibility by three investigators (AMS, DHA, MFH) independently. Abstracts of studies with favorable titles were reviewed. Studies that appeared from the abstract to be unrelated to the topic were excluded at this point. The reference lists of the full-text papers were screened for further relevant studies. The full-text papers were then screened by the above-mentioned three investigators separately and together according to predefined inclusion and exclusion criteria**.** Authors identified to have undertaken research related to the consequences of FPM extraction were contacted with a request for access to their unpublished data.

### Eligibility criteria

The following inclusion criteria were applied: a clinical trial, case-control, cross-sectional or cohort study design; patient age 5 years (time when FPM eruption starts) to 15 years (time of complete eruption of second permanent molar [SPM]) [6] at the time of extraction; and extraction of FPM due to caries or hypomineralization. The exclusion criteria were: other study design, such as a case report; patient aged older than 15 years or younger than 5 years [6] at the time of extraction; extraction of a tooth other than the FPM; and extraction of FPM as an orthodontic treatment. One study in the Turkish language [7] was translated to English by a native speaker of the Turkish language.

### Data extraction

Data were extracted from eligible papers by the three investigators working independently and included the following: site and country, study design, setting, duration, sample size, age of the patient population at the time of extraction, duration of follow-up, and methods used to evaluate the consequences of early extraction of the FPM. Only information specifically related to our research was extracted from the eligible articles.

### Meta-analysis

A meta-analysis was carried out to compare the frequency of spontaneous space closure following FPM extraction in the maxilla with that of the mandible using Review Manager Software (Rev Man 5.1, Cochrane Collaboration). The Mantel-Haenszel method was used to combine the studies for calculation of summary odds ratios and 95% confidence intervals [8]. To decide whether the results of the separate studies could be combined meaningfully, a statistical test of homogeneity was carried out. Based on the chi-square test, an inconsistency coefficient (*I*^2^ statistic) was computed where a value of more than 50% indicated moderate heterogeneity and a value of more than 75% indicated high heterogeneity [9]. The odds ratios were pooled with a random effects model for heterogeneous studies. Odds ratios with their 95% confidence limits for the individual studies and a summary estimate of effect were graphically displayed in a forest plot.

When possible, we calculated odds ratios and confidence intervals for extraction and non-extraction groups in studies missing this information. The chi-square statistic and *p*-value were calculated to compare the upper and lower arch space closure values in studies where a relationship between them was shown.

### Strengths and limitations of the included studies

The three investigators evaluated the strengths and limitations of the considered articles separately and then discussed them together. Any disagreement was resolved by consensus. The strengths and limitations of the studies were evaluated according to the STROBE (Strengthening the Reporting of Observational Studies in Epidemiology) checklist. This checklist consists of 22 items that should be reported in the title, abstract, introduction, methods, results and discussion sections of published studies, and used to assess the main observational studies, i.e., cohort, case-control and cross-sectional studies [10]. All reported items, especially in the methods and results sections of the included articles, were considered a strength of the study, while all missing items were considered a weak point. The strength of each article was scored according to the STROBE checklist: 1–7 (poor strength), 8–15 (moderate strength), and 16–22 (high strength). We also used the system devised by Shekelle et al. to grade the evidence and classify the strength of the recommendations in the included articles [11] (Additional file 1).

## Results

### Study selection

**Fig. 1:**
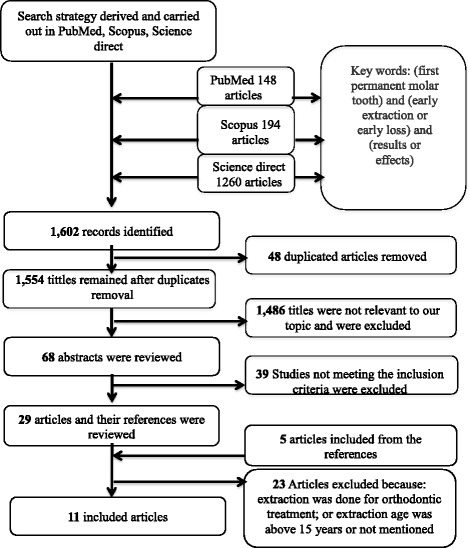
Flow diagram of study selection process

Our search strategy yielded 1602 hits, comprising 148 in PubMed, 194 in Scopus, and 1260 in Science Direct. Duplicates were removed, leaving 1554 titles. Of these titles, 68 studies were approved for evaluation; their abstracts were screened and those that were beyond the scope of our research were excluded, leaving 29 studies. References of full-text articles were checked for related articles. Five articles were added from the references. The full texts of these studies were acquired and screened according to their inclusion and exclusion criteria. The remaining articles were excluded due to either orthodontic treatment or older age at the time of extraction. No additional information was obtained by contacting authors for their unpublished data. The final result was eleven articles (Fig. 1).

### Study characteristics

Eight of the included studies were cross-sectional [12–19], two were case-control [7, 20], and one was prospective and observational [21]. One study [12] was excluded because the data for malocclusion as an outcome of FPM extraction were invalid; in addition, the criteria used to classify normal and abnormal malocclusion were not uniform or acceptable. Table 1 shows the characteristics and outcomes of the included studies.

**Table 1:** Characteristics and outcomes of included studies

Reference	Site/Country	Duration	Study design	Sample size		Age at time of extraction	Age at time of evaluation	Method of assessment	Findings		*P*-value or OR and CI
				Extraction group	Non-extraction group				Extraction group	Non-extraction group	
Effects on post extraction space											
Telli and Aytan, 1989 [7]	Turkey	1988–1989	Case-control Split-mouth	40 (T)	28 (T)	9.5 years	10.5 Years	Comparison of panoramic X-rays at extraction and a year later Comparison of cephalometric radiograph angles and distances at extraction and a year later (Angles: between long access of the SPM or second premolar and Frankfort horizontal plane in the maxilla and the occlusal plane in the mandible) (distances: between the distal surface of the upper SPM or second premolar and pterygomaxillary fissure in the maxilla and the distance between the lower SPM or second premolar and the ramus of the mandible)	Mean change in maxillary SPM angle and distance, 15.65° and 0.18 mm, respectivelyMean change in maxillary second premolar angle and distance, − 5.68° and − 2.58 mm, respectively Mean change in mandibular SPM angle and distance, 4.62° and 3.83 mm, respectively Mean change in mandibular second premolar angle and distance, − 4.63° and − 1.82 mm, respectively	Mean change in maxillary SPM angle and distance, − 2.40^o^ and 0.10 mm, respectivelyMean change in maxillary second premolar angle and distance, 2.33° and 0.53 mm, respectively Mean change in mandibular SPM angle and distance, 0.96° and 1.15 mm, respectively Mean change in mandibular second premolar angle and distance, 5.08° and 0.54 mm, respectively	*P* < 0.05* for change in extraction group; *P* > 0.05 for change in non-extraction group *P* < 0.05* for change in extraction group, *P* > 0.05 for change in non-extraction group *P* < 0.05* for change in extraction group, *P* > 0.05 for change in non-extraction group *P* < 0.05* for change in extraction group, *P* > 0.05 for change in non-extraction group
Jälevik and Möller, 2007 [14]	Specialist Clinic of Pedodontics, Sahlgrenska University Hospital, Mölndal, Department of Pedontontics, Faculty of Odontology, Göteborg University, Sweden	NA	Cross-sectional	27 subjects (16 girls and 11 boys) 66 (T)	NA	Median age 8.2 (range 5.6–12.7) years	Median age 13.9 (range 12.1–19) years	Eruption of permanent dentition, and space closure were documented by: Panoramic X-ray Dental casts Bitewings	23/27 (85.2%) subjects had spontaneous closure 52/66 (78.8%) (T) 31/38 (81.6%) Spontaneous space closure, maxilla 21/28 (75%) Spontaneous space closure, mandible	NA	Difference between mandible and maxilla *P* = 0.518 χ^2^ = 0.42 OR: 1.48 CI: (0.45, 4.83)
Rãducanu et al., 2009 [16]	Paediatric Dentistry Department, Dental Medicine Faculty of the UMF Carol Davila, Bucharest, Romania	2001–2007	Cross-sectional,hospital-based	17 subjects 22 (T) Six maxillary and 16 mandibular	NA	9–15 years	NA	Intraoral examination using dental mirror and graduated probe to evaluate post-extraction space	10/22 (45.5%) with spontaneous space closure 2/6 (33.3%) with maxillary spontaneous space closure 8/16 (50%) with mandibular spontaneous space closure	NA	Difference between mandible and maxilla *P* = 0.024* χ^2^ = 5.1 OR = 0.50 CI (0.07, 3.55)
Teo et al., 2013 [18]	Paediatric DentistryDepartment in a London-based dental hospital, UK	2008–2013	Cross-sectional Hospital based	63 subjects 236 (T) 127 SPM at Demirjian stage E *** (63 maxillary, 64 mandibular)	NA	7–13 (mean 8.9) years	Mean age 13.7 years	Panoramic X-ray to assess Demirjian’s developmental stages of SPM and to assess space closure between the contact point of the second premolar and the SPM using a ruler	101/127 (79.5%) spontaneous space closure 59/63 (94%) had spontaneous space closure in maxilla 42/64 (66%) had spontaneous space closure in mandible When SPM at stage E***)	NA	Difference between mandible and maxilla *P* = 0.0001* χ^2^ = 15.32 OR = 7.73 CI (2.48, 24.07)
Rahhal, 2014 [21]	Arab-American University Clinic, Jenin, Palestine	NA	Prospective observational study	52 (T) (maxillary FPM)	NA	10.5 years	NA	Panoramic X-ray to assess spontaneous space closure	44/52 (84.6%) maxillary spontaneous space closure	NA	NA
Teo et al., 2015 [17]	Dental Hospital, London, UK	2010	Cross-sectional, hospital-based	66 subjects 94 (T) (mandibular FPM) (71 SPM at stage E** and 23 at stage F**)	NA	Mean age 9.2 years	11–17 (mean 13.8) years	At extraction time: Panoramic X-ray to assess Demirjian’s developmental stages of SPM At recall: clinical examination with periodontal probe placed occlusally between each tooth distal to the canine to assess space closure	54/94 (57.4%) mandibular spontaneous space closure^$^ Stage E**: 41/71 (58%) mandibular spontaneous space closure^$^ Stage F**: 13/23 (56.5%) mandibular spontaneous space closure^$^	NA	NA
Effect on third molar development and eruption											
Ay et al., 2006 [13]	Department of Oral and Maxillofacial Surgery, Cumhuriyet University, Sivas, Turkey	1997–2004	Cross-sectional, hospital-based	107 subjects (unilateral extraction of mandibular FPM) 107 (T)	107 subjects 107 (T)	< 16 years	18–40 (mean 25.69) years	(Comparison of extraction and non-extraction sides in the same patient) Panoramic X-ray to assess state of impaction and impaction depth of third molars using Pell and Gregory classification and to assess third molar angulation	77/107 (72%) third molars in Class I ramus relationship^#^ 82/107 (76.6%) Class A impaction depths^^^78/107 (81.3%) in vertical positions	20/107 (18.7%) third molars in Class I ramus relationship^#^ 50/107 (46.7%) Class A impaction depths^^^37/107 (34.6%) in vertical positions	Difference between extraction and non-extraction group *P* < .001* P < .001* P < .001*
Yavuz et al., 2006 [19]	Department of Orthodontics, Dental Faculty, Atatürk University, Erzurum, Turkey	NA	Cross-sectional (comparison of extraction and non-extraction sides in the same patient), hospital-based	165 (T) 165 subjects	165 (T)165 subjects	< 12 years	13–18 (mean 15.35) years	Panoramic X-ray to assess development and eruption of third molars by measuring the vertical distances between the mesiobuccal cusp tips of the third molar and occlusal plane Dental casts to assess eruption of third molar when part of the crown is piercing the gingival tissues	28/165 (17%) third molar erupted	11/165 (6.6%) third molar erupted	*P* < 0.05*OR: 2.86*,***CI: 1.37, 5.96
Caries and/or filling of adjacent teeth											
Oliver et al., 1988 [15]	Schools of South Glamorgan, UK	1981–1984	Cross-sectional, disproportionate stratified sampling, schools-based	Occlusal384 (S) Proximal 415 (S)	Occlusal4910 (S) Proximal 5038 (S)	11–12 years (in 1980)	15–16 years (in 1984)	Intraoral examination to record caries using a two numeric code system: a tooth description code, and a surface description code Bitewings to supplement the clinical diagnosis of proximal caries	Occlusal 131/384 (34.1%) carious Proximal 33/415 (7.9%) carious	Occlusal 1153/4910 (23.5%) carious Proximal 771/5038 (15.3%) carious	*P* < 0.001* OR: 1.64*,*** CI: 1.35, 2.1*,*** OR: 0.48 CI: 0.33, 0.69
Effects on incisors											
Normando and Cavacami, 2010 [20]	Private clinics	NA	Case-control (matched for gender and age)	34 (P) (bilateral extraction of mandibular FPM)	34 (P)	≥11 years*	Non-extraction: 16–26.2 years Extraction 16–36 years	Analysis of lateral cephalometric X-rays from routine orthodontic records	1.NB (mean 23.2^o^) lingual tipping	1.NB (mean 28.4^o^) lingual tipping	*P* = 0.004*
Effects on skeletal development											
Normando and Cavacami, 2010 [20]	Private sector	NA	Case-control (matched for gender and age)	34 subjects (bilateral extraction of mandibular FPM)	34 subjects	≥11 years*	Non extraction: 16–26.2 years Extraction 16–36 years	Analysis of lateral cephalometric X-rays from routine orthodontic records	Mean GnSN 65.2^o^ Counter-clockwise rotation of occlusal plane (mean 5.6^o^) Lower anterior face height (mean 68.6 mm)	Mean GnSN 67.2^o^ Counter-clockwise rotation of the occlusal plane (mean 12.6^o^) Lower anterior face height (mean 70.8 mm)	*P* = 0.05* P = 0.0001* P = 0.048*

Notes: (−), decrease in value; *statistically significant (*P* ≤ 0.05); **according to Demirjian: stage E of dental development indicates early bifurcation development, stage F indicates late bifurcation development; ***this value was calculated by the authors according to the numbers mentioned in the study; ^#^according to Pell and Gregory classification of state of impaction in relation to the ramus, class I described as the crown is near the anterior border of the ramus^; ^^according to the Pell and Gregory classification of depth of impaction, class A described as the occlusal surface of the impacted tooth being level or nearly level with the second molar; ^$^described by the author as having contact-point displacements less than 1 mm

*Abbreviations: S* surfaces of teeth, *T* teeth, *FPM* first permanent molar, *SPM* second permanent molar, *OR* odds ratio, *CI* confidence interval, *1.NB*. lower incisor to nasion-B-point angle, *GnSN* gnathion to sella-nasion angle, *NA* value not applicable

### Outcomes

The consequences of early FPM extraction reported in the selected papers were as follows: effects on post extraction space; effects on development and eruption of the SPM and third molar; caries and/or fillings in adjacent teeth; effects on incisors; and effects on skeletal development.

### Effects on post extraction space

Six articles reported changes in the post extraction space. One was a case-control, split-mouth study [7] that analyzed linear and angular changes in the SPM and second premolar adjacent to the extraction site in cephalometric radiographs. The FPM was extracted at a mean patient age of 10.5 years. The investigators measured the angles between the long access of the SPM or second premolar and Frankfort horizontal plane in the maxilla and the occlusal plane in the mandible. They also measured the distances between the distal surface of the upper SPM or second premolar and the pterygomaxillary fissure in the maxilla and the distance between the lower SPM or second premolar and the ramus of the mandible. They reported that the FPM extraction space was closed mostly by the SPM rather than by the second premolar. SPM angles and distances increased by 15.65° and 0.18 mm, respectively, in the maxilla and by 4.62° and 3.83 mm in the mandible, while second premolar angles and distances decreased by 5.68° and 2.58 mm in the maxilla and 4.63° and 1.82 mm in the mandible when compared with the angles and distances before extraction. The results also showed a significant change in the means of all angles and distances on the extraction side after one year compared with readings at the time of extraction (*P* < 0.05). This comparison was performed by superimposition of lateral cephalometric radiographs. However, no statistically significant difference in any of the readings was found on the non-extraction side (*P* > 0.05) [7].

Jälevik and Möller [14] investigated the effect of extraction of hypomineralized FPMs in a group of children aged 5.6–12.7 years. They found that the overall rate of space closure was 85.2%. This higher rate was more frequent in the maxilla (81.6%) than in the mandible (75%), but the difference was not statistically significant (*P* = 0.518).

Rãducanu et al. [16] reported that post-extraction migration occurred in the following ways: over-eruption of opposing teeth, horizontal migration of neighboring teeth, space reduced by tipping, dual drift (horizontal and vertical), or complete space closure. The age at which FPM extraction was performed had a significant influence on the post-extraction space in both the maxillary (*P* = 0.02) and mandibular (*P* < 0.001) arches. The results of this research confirmed that spontaneous space closure does not occur in children who undergo FPM extraction after the age of 11 years. The authors reported that the rate of space closure in the mandible (50%) was significantly greater than that in the maxilla (33.3%; *P* = 0.024).

Teo et al. [18] evaluated spontaneous space closure after FPM extraction in children aged 7–13 years. They classified space closure into five categories. The first category was complete space closure between the contact points of the SPM and the second premolar. However, the other four categories had remaining space ranging from 1 mm to 5 mm or/and angulation or rotation of the SPM or second premolar. Complete space closure in the upper arch was significantly (*P* < 0.001) more likely to be achieved than in the lower arch (in 94% versus 66% of cases, respectively).

Rahhal [12, 21] studied the timing of extraction of severely decayed upper FPMs. In his sample of children (mean age 10.5 years) who underwent extraction of an upper FPM, Rahhal found that 84.6% of upper SPMs had complete space closure without any orthodontic intervention and only 15.4% of upper SPMs erupted 1 mm distal to the second premolar.

In a more recent study, Teo et al. [17] reported on space closure in a group of children of mean age 9.2 years at the time of extraction. They studied three confounding radiographic factors related to early lower FPM extraction and its relationship to space closure (Table 1). The three factors were: “1, the second premolar is engaged in the bifurcation of the second primary molar; 2, the SPM is mesially angulated in relation to the FPM; and 3, presence of the third molar”. The presence of all three factors was associated with significantly better space closure (*P* < 0.001), and a combination of the second and third factors resulted in the most favorable outcomes (*P* < 0.001).

**Fig. 2:**
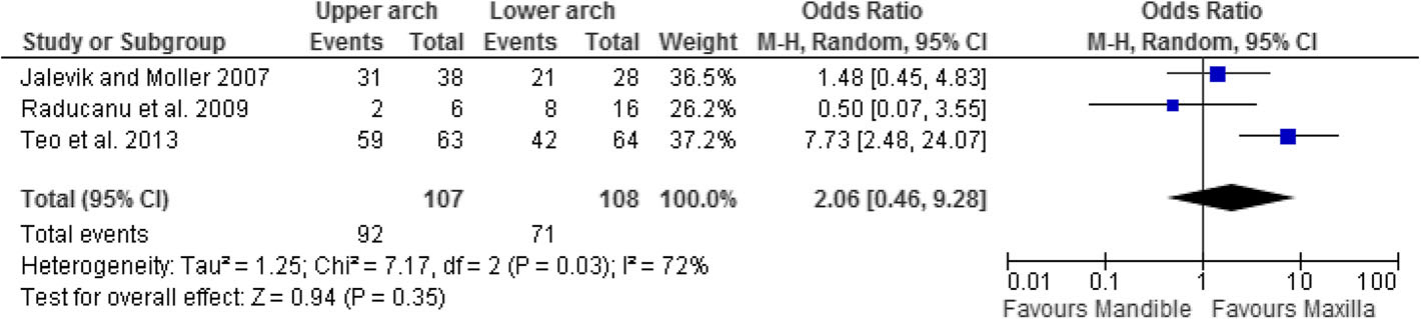
Forest plot for meta-analysis of the comparison between post extraction space closure in the upper and lower arches

A meta-analysis was performed for the three studies [14, 16, 18] that reported space closure in both arches separately to compare the frequency of spontaneous space closure of the SPM in the maxilla with that in the mandible. Space closure was more likely to occur in the maxilla than in the mandible, but the difference was not statistically significant (odds ratio 2.06, 95% confidence interval 0.46–9.28; *P* = 0.35). The heterogeneity between the three studies was moderate (I^2^ = 72%) and statistically significant (*P* = 0.03). One study found that space closure was significantly greater in the maxilla than in the mandible (94% and 66%, respectively; *P* = 0.0001) [18]. The second study also found that space closure was greater in the maxilla than in the mandible (81.6% and 75%, respectively) but the difference was not statistically significant (*P* = 0.518) [14]. In contrast, the third study found that space closure was significantly greater in the mandible than in the maxilla (50% and 33.3%, respectively; *P* = 0.024) [16]. All these results were illustrated in a forest plot (Fig. 2).

### Effect on development and eruption of the second permanent molar

A paper by Telli and Aytan [7] discussed the effect of FPM extraction on the development and eruption of the SPM. They observed that root closure and eruption of the SPM were accelerated on the extraction side when compared with the non-extraction side according to panoramic X-ray (Table 1).

### Effect on development and eruption of the third molar

Ay et al. [13] studied the changes in angle and position of the mandibular third molar after unilateral mandibular FPM extraction in a group of children aged younger than 16 years. They used the Pell and Gregory classification for the third molar in relation to the mandibular ramus and its depth of impaction as measured on panoramic X-ray [22]. This classification was described as follows: “Class I, the crown is near the anterior border of the mandibular ramus; Class II, the crown is one-half covered by the ramus; Class III, the crown is completely within the mandibular ramus” (Additional file 2) and as “Class A, the occlusal surface of the impacted tooth is level or nearly level with the SPM; Class B, the occlusal surface is between the occlusal plane and the cervical line of the SPM; Class C, the occlusal surface is below the cervical line of the second molar” (Additional file 3). Angulation of the third molar was measured between the occlusal plane of the first and second premolars and the occlusal surface of the third molar. The angles were classified as follows: vertical, < 11°; mesioangular or distoangular, 11°–70°; and horizontal, > 70°. Ay et al. found that 72% of third molars on the extraction side had their crowns near the anterior border of the mandibular ramus versus 18.7% on the non-extraction side. In addition, 76.6% of third molars on the extraction side had their occlusal surfaces at or near the level of the SPM compared with 46.7% on the non-extraction side. They also found that 81.3% of third molars were in a vertical position on the extraction side versus 34.6% on the non-extraction side. These results indicate that third molars on the extraction side underwent accelerated eruption when compared with the non-extraction side. The difference between the extraction and non-extraction sides was consistently statistically significant (*P* < 0.001) [13].

Yavuz et al. [19] studied the effects of early FPM extraction (before the age of 12 years) on development and eruption of the third molar in adolescents with mean age of 15.35 years. They reported that 17% of third molars had erupted on the extraction side compared with only 6.6% of third molars on the contralateral side; the difference was statistically significant (*P* < 0.05; Table 1).

### Caries and/or filling of adjacent teeth

Oliver et al. [15] studied the relationship between FPM extraction and the prevalence of caries and/or filling of adjacent teeth. The extractions occurred at the age of 11–12 years. The authors found significantly fewer decayed and/or filled proximal surfaces in arches with a missing FPM (7.9%) than in arches where the FPM was present (15.3%; *P* < 0.001). However, there was a significantly greater percentage of decayed and/or filled occlusal surfaces in the adjacent SPM and premolars in the arches with an extracted FPM (34.1%) when compared with arches in which the FPM was present (23.5%; *P* < 0.001; Table 1).

### Effects on incisors

Normando and Cavacami [20] discussed the effects of FPM extraction on the incisors in subjects aged approximately 11 years. Bilateral loss of the lower FPM resulted in pronounced lingual tipping (*P* = 0.004) and retrusion (*P* = 0.03) of the mandibular incisors. However, there was no pronounced effect on inclination of the maxillary incisors or their anteroposterior position (Table 1).

### Effects on skeletal development

Normando and Cavacami [20] also discussed the effects of FPM extraction on the skeleton. A decrease in the gnathion to sella-nasion angle, (i.e., the point indicating anteroinferior growth of the mandible) (*P* = 0.05), counter clockwise rotation of the occlusal plane (*P* = 0.0003), and a mild decrease in lower anterior face height (*P* = 0.048) occurred as a result of bilateral loss of the lower FPM. There was no significant effect on the maxillomandibular relationship in the anteroposterior direction (Table 1).

### Ideal time for FPM extraction

Two of the included articles [17, 18] suggested that the ideal time for FPM extraction is when development is at Demerjian stage E [23]. The stages of SPM development according to Demirjian are: “Stage D crown developed, Stage E early bifurcation, Stage F late bifurcation, and Stage G root development almost complete” [18]. In addition, one of the articles examined spontaneous space closure for SPMs that were developed to stage E or F. The spontaneous space closure rate was found to be 58% when the SPM was at stage E and 56.5% when it was at stage F. However, the difference was not statistically significant (*P* = 1.00) [17].

### Strengths and limitations of the included articles

None of the included studies reported sample size or power calculations. Only one study clearly described the study design [16]. The setting in which the study was carried out was not reported in two of the articles [7, 12, 20]. Six of the studies clearly mentioned patient age at the time of extraction and their age when the consequences of extraction were evaluated. However, two articles did not mention patient age at the time of extraction [12, 16, 20]. The sequelae were closely related to age at the time of extraction. Two articles [16, 21] did not mention age at the time of evaluation of the consequences of extraction. In addition, three of the studies did not clearly state their duration of follow-up [19–21]. Two studies did not undertake case ascertainment in their study participants [16, 20]; however, the other nine studies addressed case ascertainment by intraexaminer or interexaminer reproducibility or by using precise methods for assessment of the outcomes. Seven studies were retrospective in design and extracted information from patient records, which is considered a limitation [13, 16–18]. Interexaminer or intraexaminer reliability tests were performed in all except three articles [7, 15, 21]. Confounding factors such as age, gender, and geographic location were discussed by only one study [20] that matched patients for age, while four studies [14, 16–18] reported the age of their patients but did not match them in this regard. On the other hand, two studies [7, 15, 21] included age in their inclusion criteria. Finally, only one study reported and analyzed radiographic factors, which comprised: the second premolar being engaged in the bifurcation of the second primary molar; the SPM being mesially angulated in relation to the FPM; and the third molar being present [17] (Table 2).

**Table 2:** Strengths and limitations of included studies according to the STROBE checklist

Reference	Strengths	Limitations	Scores
Ast et al., 1961 [12]	• Clear objectives • Ascertainment of outcome and reliability of examiners were described • Extraction and non-extraction groups were matched for age	• Cross-sectional study with convenience sample • The study design was not mentioned • The sample size and power of the sample was not calculated • The study setting was not clear • Retrospective study • Age at time of extraction was not mentioned • Potential confounders and participant characteristics were not described • Weak methodology (method of assessment of molar relationship was not uniform or acceptable)	5*
Oliver et al., 1988 [15]	• The objectives were clear • The sample was stratified; however, the eligibility criteria for participant selection was not clear • Age of participants at time of extraction and evaluation of consequences were clear • Examination was carried out by one examiner • Intraexaminer reproducibility was established • Numbers of participants used for each examination was mentioned with reasons for withdrawal	• The study design was not mentioned • There was no sample size power calculation • Potential confounders were not addressed	9**
Telli and Aytan, 1989 [7]	• The objectives were clear • Age of participants at time of extraction and evaluation of consequences were clear • Split-mouth (extraction and non-extraction sides in the same patient) • Method used to assess variables was powerful (superimposition of cephalometric radiographs)	• The study design was not mentioned • There was no sample size power calculation • The location from which the sample was recruited was not clear • Reason for “non-participants” was not mentioned	10**
Ay et al., 2006 [13]	• The objectives were clear • The age was clear both at the time of extraction and evaluation of consequences • The setting was clear (Department of Oral and Maxillofacial Surgery of Cumhuriyet University, Sivas, Turkey) • Participant characteristics were mentioned • The same investigator undertook all measurements, and the reproducibility of the method was tested	• The study design was not mentioned • Retrospective • The number of extracted teeth was not clear • No sample size power calculation was performed	9**
Yavuz et al., 2006 [19]	• The objectives were clear • The location of participant recruitment was clear • The age was clear both at the time of extraction and evaluation of consequences • Reliability testing was done • Extraction and non-extraction sides were in the same patient • All assessments were performed by one examiner • Intraexaminer reproducibility was established	• The study design, method for selecting the sample, and period of recruitment were not mentioned • No sample size power calculation • A retrospective study design • Potential confounders were not addressed • Follow-up duration was not clear	11**
Jälevik and Möller, 2007 [14]	• The objectives were clear • The setting and location of patient recruitment were clear • The age of participants at time of extraction and time of evaluation of consequences were clear • Methods used to assess variables were powerful (panoramic X-rays, bitewings, casts and photographs)	• The study design was not mentioned • Period of recruitment was not mentioned • A cross-sectional study design • No sample size power calculation was performed	11**
Rãducanu et al., 2009 [16]	• The objectives were clear • The study design was clear • The location and duration of participant recruitment were clear • Intraexaminer and interexaminer reliability tests were performed	• No sample size power calculation was performed • The age at extraction and at evaluation of consequences was not clear • Numbers of each category were not mentioned, only percentages. • Convenience sample • Retrospective study • Potential confounders were not addressed • Small sample size	13**
Normando and Cavacami, 2010 [20]	• The objectives were clear • Cases and controls were matched for age, gender, and location	• The study design was not mentioned • The sample size power was not calculated • The setting in which the study was performed was not mentioned • Age at extraction time was not mentioned • Retrospective study from records • Follow-up time was not clear	13**
Teo et al., 2013 [18]	• The objectives were clear • The location and duration of participant recruitment were clear • All assessments were undertaken by one examiner • Intraexaminer repeatability was done	• The study design was not mentioned • No sample size power calculation was performed • Retrospective study from records	11**
Rahhal, 2014 [21]	• The objectives were clear • The setting was clear (Arab-American University Clinic, Jenin, Palestine) • Confirmed eligible sample	• Study design was not mentioned • The sample size power was not calculated • Age at evaluation of consequences was not mentioned • Study only performed at the upper arch • No controls • Follow-up duration was not clear	6*
Teo et al., 2015 [17]	• The objectives were clear • The setting was clear (Dental Hospital, London, UK) • The age was clear both at the time of extraction and evaluation of consequences • Intraexaminer reliability was done	• The study design was not mentioned • No sample size power calculation was performed • Retrospective study design	14**

Notes: *Scores from 1 to 7 (weak strength); **scores from 8 to 15 (moderate strength)

*Abbreviation*: *STROBE* Strengthening the Reporting of Observational Studies in Epidemiology

Scoring of the included articles was done according to the STROBE checklist. All of the articles had moderate strength except for two [12, 21] that were found to have weak strength. All the included studies were Category III, i.e., yielded evidence obtained from nonexperimental descriptive studies, such as comparative studies, correlation studies, cohort studies, and case-control studies, according to the system devised by Shekelle et al. to grade evidence [11]. In addition, all articles were scored as Class C, based on category III evidence according to the method used by Shekelle et al. to classify the strength of the recommendations (Table 3).

**Table 3:** Category of evidence and strength of recommendation

Topic	Recommendation	Category of evidence	Strength of recommendation
Effects on post extraction space	Early extraction of compromised FPM leads to decrease in post extraction space	III***	C^
Effect on development and eruption of SPM	Early extraction of compromised FPM results in accelerated development and eruption of SPM	III***	C^
Effect on development and eruption of third molar	Early extraction of compromised FPM results in accelerated development and eruption of third molar	III***	C^
Caries and/or filling of adjacent teeth	Early extraction of compromised FPM causes a decrease in caries and/or fillings of proximal surfaces on adjacent teeth	III***	C^
Effects on incisors	Early extraction of compromised FPM results in lingual tipping and retrusion of lower incisors	III***	C^
Effects on skeletal development	Early extraction of compromised FPM results in counterclockwise rotation of the occlusal plane	III***	C^

Notes: ***According to the system devised by Shekelle et al. [13] to grade evidence, Category III is evidence from nonexperimental descriptive studies, such as comparative studies, correlation studies, cohort studies, and case-control studies. ^According to the system used for classifying the strength of the recommendations devised by Shekelle et al. [13], Class C is directly based on category III evidence or a recommendation extrapolated from category I or II evidence

*Abbreviations: FPM* first permanent molar, *SPM* second permanent molar

## Discussion

Our systematic review included eleven articles that fulfilled our inclusion criteria. These papers discussed several dental and skeletal consequences related to early extraction of compromised FPM. A meta-analysis was carried out to compare the differences in spontaneous space closure between the upper and lower arches post extraction.

In one report, the principal indication for extraction of FPM was extensive caries (70%) followed by hypomineralization of the molar incisors (11%) [24]. Accordingly, in our systematic review, we included studies that addressed the consequences of early extraction of “compromised FPMs”, i.e., FPMs with severe caries or hypomineralization.

The studies included in our systematic review showed that spontaneous space closure was in the range of 45.5%–85.2%. To investigate the reason behind this wide range, we assessed the strengths and limitations of each study. In addition, we subgrouped the samples according to the site of extraction (maxilla or mandible) and carried out a meta-analysis to compare them.

The highest number of spontaneous space closures was reported by Jälevik and Möller [14], who used powerful methods to assess the eruption of the permanent dentition, and space closure, including panoramic X-rays, bitewing X-rays, casts, and photographs. In contrast, the smallest number was mentioned by Rãducanu et al. [16]. Their study had the limitations of a small sample size and a wide subject age range, which may have contributed to the low overall space closure rate.

Overall, the spontaneous space closure rate ranged from 33.3% to 94% in the maxilla and from 50% to 75% in the mandible. The smallest space closure (33.3%) in the maxilla was reported by Rãducanu et al. [16]. As mentioned above, that study had the limitations of a small sample size and subjects who varied widely in age, which may explain their low space closure rate for the maxilla. On the other hand, the largest space closure (94%) for the maxilla was reported by Teo et al. [18]. These investigators included a larger sample size and patients with SPMs at the early root bifurcation stage, which could explain their high space closure rate for the maxilla. Notably, we did not compare rates of post extraction space closure at other stages of root formation because the data needed to do this were missing.

In the recent study by Teo et al. [17], performed in 2015, the three aforementioned confounding radiographic factors that could affect post extraction space closure were analyzed from panoramic radiographs. The presence of these factors was associated with significantly better space closure. The combination of a mesially angulated SPM in relation to the FPM and the presence of the third molar resulted in the most favorable outcomes. Eichenberger et al. [25] also agreed that the presence of third molars might have a positive effect on spontaneous space closure in the mandible. However, these studies were performed only for the mandibular arch, so the maxilla and mandible could not be compared.

Regarding patient age at the time of extraction, Rãducanu et al. [16] reported that FPM extractions were performed after the age of 11 years in most children and spontaneous post extraction space closure was rarely achieved [16]. Albadri et al. [24] reported similar findings, in which children who underwent FPM extractions were older than the age allowing optimal spontaneous space closure.

Telli and Aytan [7] investigated linear and angular measurements using lateral cephalometric radiographs and reported that the FPM extraction space was closed mostly by the SPM rather than by the second premolar. These authors also found a statistically significant change in the means of all angles and distances on the extraction side after one year when compared with readings at the time of extraction. However, they did not compare this outcome with the distance needed for space closure. In addition, they did not report on how many patients achieved complete closure or the standard deviation of the mean distance and angulation, so we were not able to carry out any further analysis.

Three studies reported differences in the amount of space closure between the maxilla and the mandible [14, 16, 18] and were subjected to meta-analysis. Jälevik and Möller [14] and Teo et al. [18] reached the same conclusion i.e., that space closure after extraction of a compromised FPM was better in the maxilla than in the mandible. However, only Teo et al. [18] showed a significant difference between the maxilla and the mandible [18]. In contrast, another study reported that complete space closure was more likely to be achieved in the mandible than in the maxilla [16].

The difference in the eruption pathway between the maxilla and mandible could be the reason that the maxilla showed better space closure. The apex of the maxillary SPM is usually mesially placed in relation to the crown, and during spontaneous space closure, the crown tends to tip mesially into a more satisfactory position [26]. However, complete space closure was achieved in only 66% of cases where the mandibular FPM was extracted when the SPMs were at the early bifurcation stage. As explained by Gill et al. [5], extraction of the mandibular FPM before or after this stage will not result in complete space closure. Extraction before early bifurcation may result in distal drifting, tilting and rotation of the unerupted second premolar because it lies in an unrestrained position apical to the roots of the second deciduous molar. If the FPM is extracted during or after eruption of the SPM, complete space closure is usually not achieved. This could be due to the occlusal forces that encourage lingual tilting of the SPM, given that the lingual plate is thinner than the buccal plate of alveolar bone, which in turn affects the likelihood of complete space closure.

One study reported that 84.6% of maxillary SPMs had complete space closure without any orthodontic intervention; however, this study had some limitations, including no mention of the age at evaluation of consequences, lack of controls, and an unclear follow-up duration [21]. Therefore, we cannot rely on these results; in addition, the study was performed only on the maxilla, so a comparison between the maxilla and the mandible cannot be made.

One article discussed the effect of extraction of the FPM on the development and eruption of the SPM. On analysis of panoramic X-rays, the authors found that root closure and eruption of SPM were accelerated on the extraction side when compared with the non-extraction side [7]. However, these results were not subjected to statistical analysis, which is considered a weakness of this study. Further studies are needed to confirm these findings.

The prevalence of third molar impaction worldwide has been reported to be 24.40% [27]. Further, in 2016, Hatem et al. [28] reported that 70% of third molars were classified as impacted. Al-Anqudi et al. [29] found that 54.3% of patients had at least one impacted third molar. Therefore, extracting posterior teeth can increase the eruption space for third molars by mesial movement of the molars [13]. Third molars observed on the extraction side were closer to the anterior border of the mandibular ramus, the occlusal surface was level or nearly level with the SPM, and more vertically positioned when compared with the non-extraction side, which indicates acceleration of third molar eruption. This is due to the fact that mandibular FPM extraction increased the space for third molar eruption and movement into a better position [13]. The same investigator made all the measurements, and the reproducibility of the method was tested, which is considered a strength of this study. However, the exact number of teeth extracted was not stated.

In addition, it was reported that the development and eruption of the third molars on the FPM extraction sides were significantly accelerated compared with the contralateral sides, as evaluated by panoramic X-rays and dental casts. The third molars on the extraction sides in 96 of 131 cases (73.3%) were closer to the occlusal plane than the third molars on the contralateral sides [19]. Gill et al. [5] reported similar results, i.e., a 90% chance of successful third molar eruption after FPM extraction compared with a 55% chance following premolar extraction. Most third molars had a tendency to erupt early and establish a good contact relationship with the SPM.

According to panoramic X-rays and clinical examination, significantly fewer decayed and/or filled proximal surfaces were found in arches with missing FPMs (8% when FPMs were missing and 15% when they were present). This was due to ease of cleaning, accessibility for application of fluoride, and less accumulation of plaque. In contrast, significantly more decayed and/or filled occlusal surfaces were found in arches with FPM extraction in the adjacent SPMs and premolar teeth than arches in which the FPM was present (34% and 24%, respectively). SPMs had more carious or restored occlusal surfaces than second premolars [15]. Extraction of FPM is not the main etiological factor for the development of occlusal caries. However, children requiring FPM extraction were at higher risk for developing occlusal decay in all teeth.

Using cephalometric measurements, Normando and Cavacami [20] found that pronounced lingual tipping and retrusion of the mandibular incisors had resulted from bilateral loss of the lower FPM. While there was no pronounced effect on the inclination of the maxillary incisors nor their anteroposterior position due to bilateral extraction of the lower FPM [20]. Extraction of the lower FPM results in increased overbite and overjet due to lingual inclination of the lower incisors [30].

After comparing lateral cephalometric measurements between a control group and a group with bilateral loss of the FPM, Normando and Cavacami [20] reported several findings. A decrease in the gnathion to sella-nasion angle, counter clockwise rotation of the occlusal plane, and a mild decrease in lower anterior face height occurred as a result of bilateral loss of the lower FPM. These findings were due to the common clinical observation of loss of the vertical dimension resulting from bilateral loss of the FPM. No significant effect was found with regard to the maxillomandibular relationship in the anteroposterior direction analyzed using different linear and angular measurements. Notably, the mandible was more affected than the maxilla, with regard to both effects on the incisors and effects on skeletal development [20]. Nevertheless, this report did not mention the ages at which FPMs were extracted, the duration of follow-up, nor the study setting, so its findings need further clarification.

Malocclusion is a well-known problem that could affect the developing dentition following premature loss of primary or permanent teeth [31]. Malocclusion presents a health issue, as it might negatively influence the quality of life of young patients [32]. Ast et al. [12] claimed that malocclusion was more common in the FPM extraction group (97%) than in the non-extraction group (70%), suggesting that malocclusion could result from early tooth extraction. They also found that ingestion of fluoridated water from birth onwards in children resulted in significant protection against dental caries, and significantly lower rates of loss of permanent teeth, specifically FPMs, and consequently less malocclusion when compared with children who did not ingest fluoridated water at the optimum concentration [12]. Notably, this retrospective study did not mention the design, setting, or the ages at which FPMs were extracted. In addition, the method used to address malocclusion was not described and the method used to assess the molar relationship was neither uniform nor acceptable. Therefore, the results of this study cannot be considered reliable.

The ideal time for FPM extraction is when the SPM is at the early bifurcation stage. According to our systematic review, extraction of compromised FPM has three sequelae, i.e., space changes, effects on the incisors, and effects on skeletal development. These sequelae are more related to the mandible than to the maxilla [14, 18, 20]. Spontaneous space closure was more difficult to achieve in the mandible than in the maxilla [14, 18]. Lingual tipping and retrusion of the lower incisors and a mild decrease in lower anterior face height were also reported [20]. As a result, the decision to extract the FPM is more critical in the mandible than in the maxilla. Nevertheless, we do not hesitate to extract in the maxilla.

Our systematic review has some limitations. The included studies were observational (cross-sectional and case-control) and seven were retrospective. There is a need for prospective studies to confirm the consequences of FPM extraction. Other variables that affect the consequences of FPM extraction should also be tested.

## Conclusions and recommendations

The outcomes of early extraction of FPM include a decrease in post extraction space, accelerated development and eruption of the SPM and third molar, a decrease in caries and/or fillings on proximal surfaces of adjacent teeth, lingual tipping and retrusion of incisors, and counter clockwise rotation of the occlusal plane. Prevention of caries in children would avoid these consequences. Where extraction of the FPM is unavoidable, the ideal time to extract should be taken into consideration using the Demirjian classification for development of the SPM rather than by chronological age. The ideal time for FPM extraction; with fewer undesirable consequences, is when the SPM is at Demirjian stage E (early bifurcation). The decision to extract a FPM in the mandible is more difficult than for one in the maxilla, because the sequelae relate more to the mandible than to the maxilla. It appears that the studies included in this review have too many weaknesses to draw sufficient evidence. Therefore, further longitudinal studies are needed to investigate the influence of age and sex on time of extraction and to compare the consequences of extraction with those of other treatment modalities in order to arrive at a decision on how to proceed in these patients. Finally, confirmation of previous outcomes should be considered, and other variables that could influence the outcome should be tested in longitudinal studies.

## Additional files

Additional file 1 Scheme devised by Shekelle et al. for classifying the evidence for and strength of study recommendations. (DOCX 192 kb)

Additional file 2 Pell and Gregory classification of third molar in relation to the mandibular ramus. Class I: the crown is near the anterior border of the mandibular ramus. Class II: the crown is one-half covered by the ramus. Class III: the crown is completely within the mandibular ramus. (DOCX 104 kb)

Additional file 3 Pell and Gregory classification according to impaction depth of third molar. Class A: the occlusal surface of the impacted tooth is level or nearly level with the second molar. Class B: the occlusal surface is between the occlusal plane and the cervical line of the second molar. Class C: the occlusal surface is below the cervical line of the second molar. (DOCX 97 kb)

## Abbreviations

ADA: American Dental Association
CI: Confidence interval
FPM: First permanent molar
GnSN: Gnathion to sella-nasion angle
NA: Value not applicable
NB: Lower incisor to nasion-B-point angle
OR: Odds ratio
S: Surfaces of teeth
SPM: Second permanent molar
STROBE: Strengthening the Reporting of Observational Studies in Epidemiology
T: Teeth
WHO: World Health Organisation

## References

[1] Ozdemir D (2013). Dental Caries : The most common disease worldwide and preventive strategies. Int J Biol.

[2] World Health Organization (WHO). Oral Health*.* World Health Organization. 2012 [Available from: http://www.who.int/mediacentre/factsheets/fs318/en/ [Accessed 15-2-2016 2016].

[3] American Dental Association (ADA). Tooth eruption. J Am Dent Assoc. 2006 [Available from: http://www.ada.org/~/media/ADA/Publications/Files/patient_58.ashx [Accessed 15–2-2016 2016].

[4] ElSheikh M, Ali A (2015). Planned extraction of first permanent molars during late childhood: a clinical note and mini-review. Dent Oral Craniofac Res.

[5] Gill DS, Lee RT, Tredwin CJ (2001). Treatment planning for the loss of first permanent molars. Dent Update.

[6] Ekstrand KR, Christiansen J, Christiansen ME (2003). Time and duration of eruption of first and second permanent molars: a longitudinal investigation. Community Dent Oral Epidemiol.

[7] Telli AE, Aytan S (1989). Changes in the dental arch due to obligatory early extraction of first permanent molars. Turk Ortodonti Dergisi.

[8] Mantel N, Haenszel W (1959). Statistical aspects of the analysis of data from retrospective studies of disease. J Natl Cancer Inst.

[9] Higgins JP, Thompson SG, Deeks JJ, Altman DG (2003). Measuring inconsistency in meta-analyses. BMJ.

[10] von Elm E, Altman DG, Egger M, Pocock SJ, Gotzsche PC, Vandenbroucke JP (2008). The strengthening the reporting of observational studies in epidemiology (STROBE) statement: guidelines for reporting observational studies. J Clin Epidemiol.

[11] Shekelle PG, Woolf SH, Eccles M, Grimshaw J (1999). Clinical guidelines: developing guidelines. BMJ.

[12] Ast DB, Allaway N, Draker HL (1962). The prevalence of malocclusion, related to dental caries and lost first permanent molars, in a fluoridated city and a fluoride-deficient city. Am J Orthod.

[13] Ay S, Agar U, Bicakci AA, Kosger HH (2006). Changes in mandibular third molar angle and position after unilateral mandibular first molar extraction. Am J Orthod Dentofac Orthop.

[14] Jalevik B, Moller M (2007). Evaluation of spontaneous space closure and development of permanent dentition after extraction of hypomineralized permanent first molars. Int J Paediatr Dent.

[15] Oliver SJ, Dummer PM, Oliver RG, Hicks R, Addy M, Kingdon A, Shaw WC (1988). The relationship between loss of first permanent molar teeth and the prevalence of caries and restorations in adjacent teeth: a study of 15-16- year-old children. J Dent.

[16] Rãducanu AFV, Herteliu C, Rãducanu M (2009). Prevalence of loss of permanent first molars in a group of Romanian children and adolescents. Oral Health Dent Manag.

[17] Teo TK, Ashley PF, Derrick D (2015). Lower first permanent molars: developing better predictors of spontaneous space closure. Eur J Orthod.

[18] Teo TK, Ashley PF, Parekh S, Noar J (2013). The evaluation of spontaneous space closure after the extraction of first permanent molars. Eur Arch Paediatr Dent.

[19] Yavuz I, Baydas B, Ikbal A, Dagsuyu IM, Ceylan I (2006). Effects of early loss of permanent first molars on the development of third molars. Am J Orthod Dentofac Orthop.

[20] Normando D, Cavacami C (2010). The influence of bilateral lower first permanent molar loss on dentofacial morfology – a cephalometric study. Dental Press J Orthod.

[21] Rahhal A (2014). Extraction timing of heavily destructed upper first permanent molars. Czas Stomatol.

[22] Pell GJ, Gregory GT (1933). Impacted mandibular third molars: classification and modified technique for removal. Dent Digest.

[23] Demirjian A, Goldstein H, Tanner JM (1973). A new system of dental age assessment. Hum Biol.

[24] Albadri S, Zaitoun H, McDonnell ST, Davidson LE (2007). Extraction of first permanent molar teeth: results from three dental hospitals. Br Dent J.

[25] Eichenberger M, Erb J, Zwahlen M, Schatzle M (2015). The timing of extraction of non-restorable first permanent molars: a systematic review. Eur J Paediatr Dent.

[26] Crabb JJ, Rock WP (1971). Treatment planning in relation to the first permanent molar. Br Dent J.

[27] Carter K, Worthington S (2016). Predictors of third molar impaction: a systematic review and meta-analysis. J Dent Res.

[28] Hatem M, Bugaighis I, Taher EM (2016). Pattern of third molar impaction in Libyan population: a retrospective radiographic study. Saudi J Dent Res.

[29] Al-Anqudi SM, Al-Sudairy S, Al-Hosni A, Al-Maniri A (2014). Prevalence and pattern of third molar impaction: a retrospective study of radiographs in Oman. Sultan Qaboos Univ Med J.

[30] Abu Aihaija ES, McSheny PF, Richardson AA (2000). Cephalometric study of the effect of extraction of lower first permanent molars. J Clin Pediatr Dent.

[31] Tinanoff N, Kliegman RM, Behrman RE, Jenson HB, Stanton BF (2011). Malocclusion. Nelson textbook of pediatrics.

[32] Dimberg L (2015). Malocclusions and Quality of life. Cross-sectional and longitudinal studies in children. Swed Dent J Suppl.

