# Supplementary material for: Consequences of early extraction of compromised first permanent molar: a systematic review
Source: BMC Oral Health. 2018 Apr 5;18:59. doi: 10.1186/s12903-018-0516-4 (PMC5887204; doi:10.1186/s12903-018-0516-4)
Supplement: Supplementary file 1 — Scheme devised by Shekelle et al. for classifying the evidence for and strength of study recommendations. (DOCX 192 kb) [file 12903_2018_516_MOESM1_ESM.docx]

**Appendices**

**Additional file 1**

Scheme devised by Shekelle et al for classifying the evidence for and strength of study recommendations


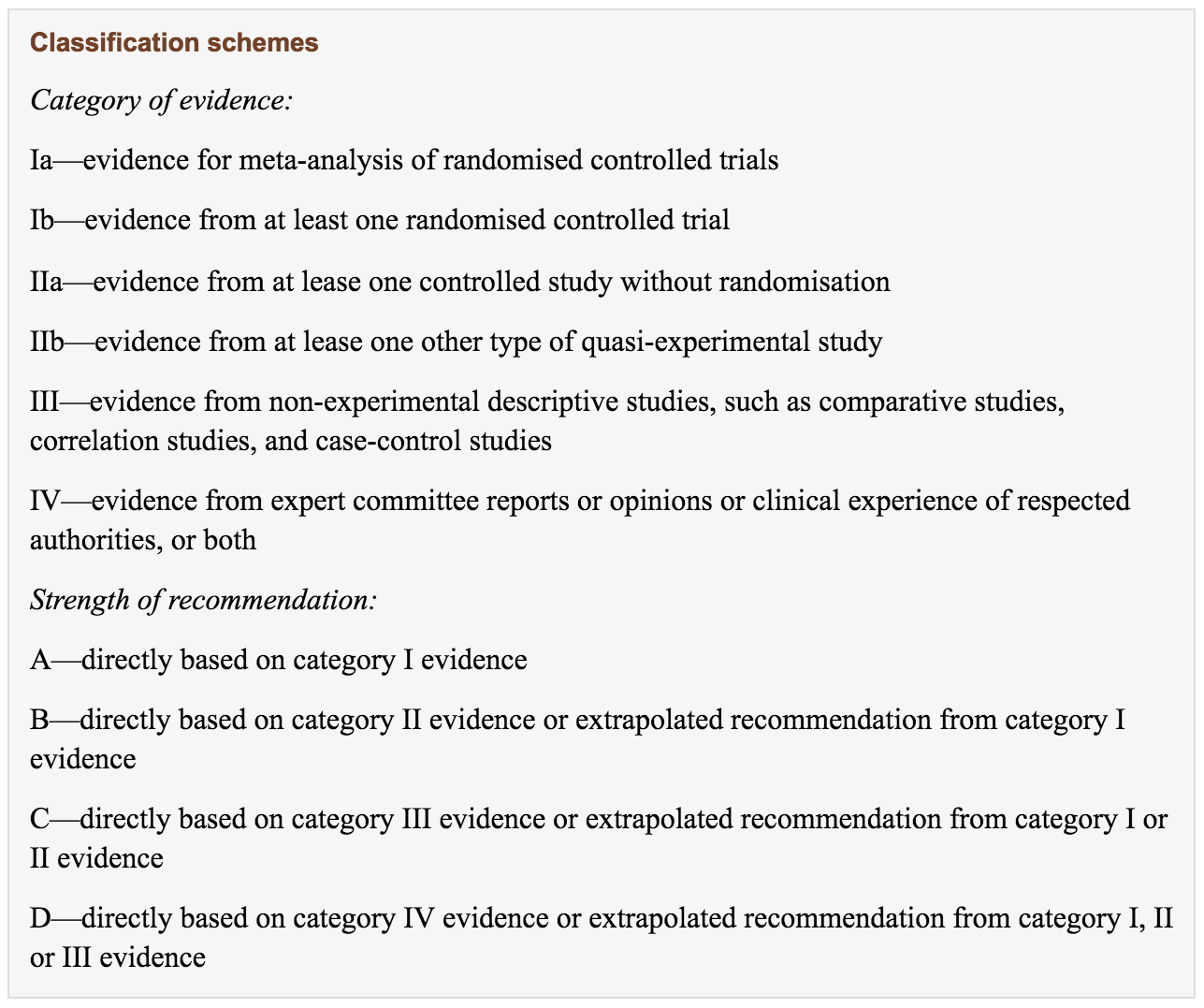
Adapted with the permission of the BMJ Publishing Group from: Shekelle PG, Woolf SH, Eccles M, Grimshaw J. Clinical guidelines: developing guidelines. *British Medical Journal (Clinical Research Ed).* 1999; **318**: 593–6.
