# Supplementary material for: Consequences of early extraction of compromised first permanent molar: a systematic review
Source: BMC Oral Health. 2018 Apr 5;18:59. doi: 10.1186/s12903-018-0516-4 (PMC5887204; doi:10.1186/s12903-018-0516-4)
Supplement: Supplementary file 2 — Pell and Gregory classification of third molar in relation to the mandibular ramus. Class I: the crown is near the anterior border of the mandibular ramus. Class II: the crown is one-half covered by the ramus. Class III: the crown is completely within the mandibular ramus. (DOCX 104 kb) [file 12903_2018_516_MOESM2_ESM.docx]

**Additional file 2**


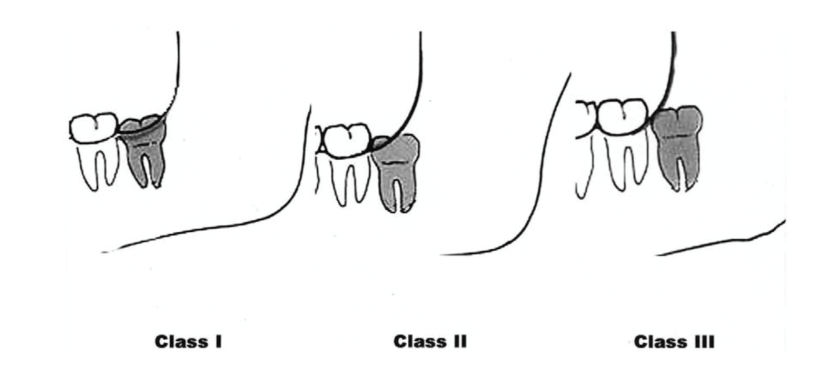
Pell and Gregory classification of third molar in relation to the mandibular ramus. Class I: the crown is near the anterior border of the mandibular ramus. Class II: the crown is one-half covered by the ramus. Class III: the crown is completely within the mandibular ramus.

Adapted with the permission of American Journal of Orthodontics and Dentofacial Orthopedics from: Ay S, Agar U, Bicakci AA, Kosger HH. Changes in mandibular third molar angle and position after unilateral mandibular first molar extraction. *American Journal of Orthodontics and Dentofacial Orthopedics*. 2006; **129**: 36–41.
