# Supplementary material for: Consequences of early extraction of compromised first permanent molar: a systematic review
Source: BMC Oral Health. 2018 Apr 5;18:59. doi: 10.1186/s12903-018-0516-4 (PMC5887204; doi:10.1186/s12903-018-0516-4)
Supplement: Supplementary file 3 — Pell and Gregory classification according to impaction depth of third molar. Class A: the occlusal surface of the impacted tooth is level or nearly level with the second molar. Class B: the occlusal surface is between the occlusal plane and the cervical line of the second molar. Class C: the occlusal surface is below the cervical line of the second molar. (DOCX 97 kb) [file 12903_2018_516_MOESM3_ESM.docx]

**Additional file 3**

Pell and Gregory classification according to impaction depth of third molar. Class A: the occlusal surface of the impacted tooth is level or nearly level with the second molar. Class B: the occlusal surface is between the occlusal plane and the cervical line of the second molar. Class C: the occlusal surface is below the cervical line of the second molar.

**
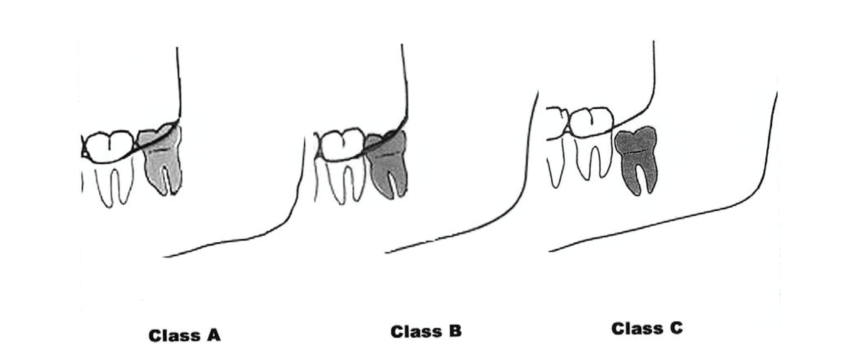
**

Adapted with the permission of American Journal of Orthodontics and Dentofacial Orthopedics from: Ay S, Agar U, Bicakci AA, Kosger HH. Changes in mandibular third molar angle and position after unilateral mandibular first molar extraction. *American Journal of Orthodontics and Dentofacial Orthopedics*. 2006; **129**: 36–41.
